# Adaptation of a Theory-Based Mobile App to Improve Access to HIV Prevention Services for Transgender Women in Malaysia: Focus Group Study

**DOI:** 10.2196/56250

**Published:** 2024-08-13

**Authors:** Kamal Gautam, Roman Shrestha, Sihlelelwe Dlamini, Belle Razali, Kiran Paudel, Iskandar Azwa, Rumana Saifi, YuHang Toh, Hazriq Justin Lim, Ryan Sutherland, Arjee Restar, Nittaya Phanuphak, Jeffrey A Wickersham

**Affiliations:** 1 Department of Allied Health Sciences University of Connecticut Storrs, CT United States; 2 Department of Internal Medicine Yale School of Medicine New Haven, CT United States; 3 Centre of Excellence for Research in AIDS Faculty of Medicine University of Malaya Kuala Lumpur Malaysia; 4 Infectious Diseases Unit, Faculty of Medicine University Malaya Kuala Lumpur Malaysia; 5 Department of Epidemiology School of Public Health, University of Washington Seattle, WA United States; 6 Institute of HIV Research and Innovation Bangkok Thailand

**Keywords:** HIV, AIDS, pre-exposure prophylaxis, PrEP, mobile health, mHealth, transgender women, Malaysia, mobile phone

## Abstract

**Background:**

Globally, transgender women have been disproportionately affected by the HIV epidemic, including in Malaysia, where an estimated 11% of transgender women are living with HIV. Available interventions designed specifically to meet transgender women’s needs for HIV prevention are limited. Mobile health, particularly smartphone mobile apps, is an innovative and cost-effective strategy for reaching transgender women and delivering interventions to reduce HIV vulnerability.

**Objective:**

This study aims to adapt a theory-based mobile health HIV prevention smartphone app, HealthMindr, to meet the unique needs of transgender women in Malaysia. We conducted theater testing of the HealthMindr app with transgender women and key stakeholders and explored barriers to transgender women’s uptake of HIV pre-exposure prophylaxis (PrEP).

**Methods:**

From February to April 2022, a total of 6 focus group (FG) sessions were conducted with 29 participants: 4 FG sessions with transgender women (n=18, 62%) and 2 FG sessions with stakeholders (n=11, 38%) providing HIV prevention services to transgender women in Malaysia. Barriers to PrEP uptake and gender-affirming care services among transgender women in Malaysia were explored. Participants were then introduced to the HealthMindr app and provided a comprehensive tour of the app’s features and functions. Participants provided feedback on the app and on how existing features should be adapted to meet the needs of transgender women, as well as any features that should be removed or added. Each FG was digitally recorded and transcribed. Transcripts were coded inductively using Dedoose software (version 9.0.54; SocioCultural Research Consultants, LLC) and analyzed to identify and interpret emerging themes.

**Results:**

Six subthemes related to PrEP barriers were found: stigma and discrimination, limited PrEP knowledge, high PrEP cost, accessibility concerns, alternative prevention methods, and perceived adverse effects. Participants suggested several recommendations regarding the attributes and app features that would be the most useful for transgender women in Malaysia. Adaptation and refinement of the app were related to the attributes of the app (user interface, security, customizable colors, themes, and avatars), feedback, and requests for additional mobile app functional (appointment booking, e-consultation, e-pharmacy, medicine tracker, mood tracker, resources, and service site locator) and communication (peer support group, live chat, and discussion forum) features.

**Conclusions:**

The results reveal that multifaceted barriers hinder PrEP uptake and use among transgender women in Malaysia. The findings also provide detailed recommendations for successfully adapting the HealthMindr app to the context of Malaysian transgender women, with a potential solution for delivering tailored HIV prevention, including PrEP, and increasing accessibility to gender-affirming care services.

## Introduction

### Background

Transgender women are disproportionately impacted by the global HIV epidemic. A recent systematic review of 94 studies on the global burden of HIV in transgender women found HIV prevalence to be at 19.9% (95% CI 14.7%-25.1%) [1]. Transgender women have some of the highest HIV burdens in Southeast Asia, including Malaysia, where the rate of new HIV infections among transgender women is increasing, with higher estimates among those engaged in sex work (12.4%) [2-6].

Available literature suggests that legal restrictions, stigma, discrimination, and limited economic and educational opportunities intersect synergistically in increasing both HIV vulnerability and prevalence among transgender women [7,8]. Sharia laws in Malaysia, which criminalize “a man posing as a woman,” subject transgender women to legal restrictions and frequent harassment, leading to arrests and abuse by religious and law enforcement authorities [9,10]. This oppressive environment, coupled with limited economic opportunities, compels many transgender women to engage in sex work, significantly increasing their risk of HIV infection through multiple sex partners, drug use, and less health-seeking behavior [11-16]. Despite this evidence, most HIV prevention and treatment interventions have either not included transgender women or have counted them as men who have sex with men (MSM). Interventions explicitly designed to meet the needs of transgender women are critical to achieving the target of ending the AIDS epidemic by 2030 [17].

The World Health Organization recommends offering HIV pre-exposure prophylaxis (PrEP) to individuals at substantial risk of infection, including transgender women, as part of a comprehensive prevention package that includes condom use, sexually transmitted infection (STI) screening, and access to early HIV diagnosis and antiretroviral therapy. Regular HIV testing as well as PrEP medication, if taken as prescribed, is a safe and effective tool for the prevention of HIV [18-20]. In Malaysia, daily oral PrEP has been available since 2017 under the National Strategic Plan to end AIDS by 2030 [21]. In 2023, the government launched a free PrEP program in selected public clinics to enhance accessibility [22]. In addition, plans are in place to introduce long-acting injectable PrEP by 2027, further expanding preventive options [23]. Despite these efforts, HIV testing and oral PrEP uptake are low among transgender women in Malaysia. One recent study conducted with 199 transgender women in Kuala Lumpur (KL), the capital city, found the lifetime HIV testing rate at 41.7% and recent HIV testing rate at 18.7% [24]. Similarly, another survey with 361 transgender women from 5 Malaysian states found that only 20% had ever heard of PrEP, and none had ever used it [7]. However, this same study found that transgender women had a high level of willingness to use PrEP (83%) if it were accessible to them [7]. While PrEP has been freely available in a few government clinics since early 2023, this low uptake is due to the legal barriers that criminalize transgender identities, making it difficult for transgender women to seek health care and disclose their gender identity and risk behaviors [9,16,25,26]. Consequently, this antitransgender sociopolitical environment limits other health services that are pertinent and salient to transgender people’s lives, pushing transgender women to take care of themselves outside the formal health care system; for example, transgender women have been documented to self-administer antibiotics and gender-affirming hormones, which may be life-threatening if not adequately monitored, due to the challenges of obtaining accessible, safe, and quality health care services [16,27].

Mobile health (mHealth), specifically mHealth apps, may be particularly useful in overcoming barriers associated with accessing traditional clinic-based care in settings where antitransgender stigma persists [28-30]. Mobile apps can offer anonymous access to health care services (eg, HIV testing, PrEP, and gender-affirming care); provide referrals; and facilitate decision-making in a private, confidential, and convenient environment [31-34]. Recent reports show that Malaysia has a high rate of internet penetration (89.5%) and smartphone use (98.9%) [35]. Among Malaysians aged 16 to 64 years, 98.9% use the internet via a smartphone and spend >9 hours daily using the internet [35]. A comprehensive mobile app that provides HIV prevention and treatment services for transgender women does not exist. However, previous studies show that transgender women in Malaysia use their smartphones to socialize and make sexual connections [7] and show high acceptability of mHealth [36]. This evidence suggests that the development of a comprehensive mobile app tailored to the needs of transgender women in Malaysia may provide a solution to their unaddressed health needs.

### Objectives

The objectives of this study were to (1) identify potential barriers to accessing and using PrEP among transgender women in Malaysia and (2) describe the findings from the first phase of adaptation of the smartphone app HealthMindr [37] through theater testing [38-40]. Theater testing focused on understanding how the HealthMindr app’s key features and functions should be adapted to ensure optimal uptake among transgender women in Malaysia.

## Methods

### Participants and Study Settings

Six focus group (FG) sessions were conducted with 29 participants: 4 FG sessions with transgender women (n=18, 62%) and 2 FG sessions with stakeholders (n=11, 38%) providing HIV prevention services to transgender women in Malaysia. FG sessions were conducted until theoretical saturation was achieved. Transgender women participants were recruited through advertisements on social media platforms, referrals from transgender advocacy organizations, and peer referrals. Interested individuals who scanned the QR code or clicked the advertisement link were redirected to Qualtrics (Qualtrics International Inc) to provide their details and contact information. Subsequently, research staff contacted eligible participants to determine whether they would like to participate in the study, providing comprehensive details about the study’s objectives and the rights of participants.

The inclusion criteria for transgender women were as follows: (1) aged ≥18 years; (2) self-reported HIV-negative status or HIV status unknown; (3) currently use an Android or iOS smartphone; (4) able to speak and understand Bahasa Malaysia; and (5) have at least 1 HIV-related sexual risk behavior in the last 6 months, including engagement in sex work, having >1 sexual partner without consistent condom use or use of PrEP, or engagement in chemsex—defined as the use of methamphetamine, mephedrone, gamma-hydroxybutyrate and gamma-butyrolactone, or 3,4-methylenedioxy-methamphetamine (commonly known as *ecstasy*) before or during sexual activity. The inclusion criteria for stakeholders were as follows: (1) aged ≥18 years; (2) currently employed as a physician, counselor, pharmacist, or staff member of a nongovernmental organization (NGO) involved in providing HIV-related services to transgender women; and (3) able to speak Bahasa Malaysia or English. Stakeholders were recruited through referrals from transgender advocacy organizations and clinics serving transgender women.

### Study Procedures

FG sessions were conducted between February and April 2022 using the Yale University–licensed videoconferencing platform, Zoom (Zoom Video Communications, Inc). FG sessions were facilitated and moderated by 2 trained research staff members. Each Zoom FG meeting was password protected, with unique invitations sent to every FG participant. Upon joining, attendees were first placed in a *waiting room*. In the waiting room, participants’ identities were verified, and they were given the option to change their screen names to pseudonyms and keep their cameras off during the FG sessions to ensure privacy and confidentiality. The participants were encouraged to provide verbal feedback or via a chat function, which was regularly monitored to capture all responses. Before the beginning of each FG session, participants completed a brief demographic and behavioral questionnaire via Qualtrics. Using a semistructured FG guide (Multimedia Appendix 1), we asked participants to discuss the key barriers to PrEP uptake and use among transgender women.

Next, one of the facilitators provided a live tour of the HealthMindr mobile app, demonstrating the app’s multiple features and functions designed to facilitate improved HIV prevention for MSM in the United States. The HealthMindr app, which is based on social cognitive theory, has shown high usability and user satisfaction [38]. Social cognitive theory posits that cognition, behavior, and environmental influences interact to impact health behavior. For the app, it specifies goal setting, self-efficacy, outcome expectations, and self-regulation as essential influences of health behavior. Current features fit the framework for several health behaviors, including making HIV testing plans, using condoms, screening for PrEP, and seeking PrEP (if eligible). For each health behavior, there are specific app features that are designed to promote goal setting, self-efficacy, outcome expectations, and self-regulation; for example, regarding HIV testing, the *Make a Plan* feature promotes goal setting, the presentation of testing options promotes self-efficacy, information about the benefits of testing promotes positive outcome expectations, and a customizable reminder promotes testing self-regulation [38].

The facilitator demonstrated how the app provides HIV testing information and reminders to test regularly, as well as searches for testing site locators. Participants were shown how users could complete monthly or on-demand behavioral risk assessments and access PrEP information. The facilitator also explained how the app allows users to order condoms, lubricants, and HIV self-testing kits. In addition, they highlighted the app’s screening features for anxiety, depression, and substance use, along with a directory of local counseling resources [37].

After the live tour, participants were asked to provide feedback on the HealthMindr app format, content, and features as well as preferences for additional features, focusing on its adaptation for transgender women in Malaysia. Each FG session, which lasted between 60 and 90 minutes, was audio recorded and stored in the Zoom cloud in a Yale-affiliated researcher’s private account, protected by 2-factor authentication and password protection. FG sessions were transcribed and translated from Bahasa Malaysia into English, with back translation conducted to ensure accuracy.

### Data Analysis

SPSS software (version 29.0; IBM Corp) was used to calculate descriptive statistics for the variables collected from the questionnaire. Qualitative data analysis was conducted using an inductive coding approach to identify themes and patterns in the FG transcripts. Three coders (SD, BR, and KP) read and reread the transcripts to gain an overall understanding and identify the initial impression of the data. During the open coding phase, the coders independently assigned codes to segments of transcript text with distinct concepts or themes. After the initial coding, 3 debriefing meetings were held between the coders, and a codebook was developed to ensure consistency through the discussion of their relevance and suitability. After the coding meetings, a final coder (KG) reviewed the draft codebook and organized the codes into root codes representing larger categories and child codes within each larger subset using an arbitration process. Dedoose software (version 9.0.54; SocioCultural Research Consultants, LLC) was used for FG data management and analysis.

### Ethical Considerations

The study was reviewed and approved by the institutional review boards of Yale University (2000030146) and Universiti Malaya (2021115-10758). The study’s purpose as well as potential benefits and risks were shared with participants, and verbal informed consent was obtained. The protocol was approved by the institutional review boards with a waiver of written documentation of consent. All transcripts were deidentified before the analysis to ensure confidentiality by removing personal identifiers, such as names, organizational affiliations, and positions. Each participant received 40 Malaysian ringgit (approximately US $10) as compensation for their time.

### Reflexivity and Positionality

The majority of the study authors (11/13, 85%) are from the Global South. Of these 11 authors, 4 (36%) are Malaysian, and several of the other authors (7/9, 78%) possess extensive knowledge and prior work experience in Malaysia. All FG sessions with transgender women and stakeholders were led by a Malaysian transgender woman researcher to ensure that nothing was lost in the interpretation and translation when the researcher conducted the FGs and in the translation of the transcripts from Bahasa Malaysia into American English. During the data analysis, the researchers involved in coding the data paid attention to their own beliefs, judgments, and practices and reflected on how these may influence their understanding of the data and management of the study. We relied heavily on the experiences of our Malaysian coauthors to align our potential differences in perspective and gain a deeper understanding of the data. All authors who identified themselves as cisgender heterosexual reflected on their power and privilege and remained reflective of all potential biases throughout the study.

## Results

### Participant Demographics

#### Overview

Participant sociodemographics, PrEP knowledge, history, concerns about using PrEP, recent sexual behavior, and smartphone and internet use are presented in Table 1.

**Table 1:** Characteristics of participants (N=29).

Variable						Values
**Transgender women (n=18)**						
	Age (y), mean (SD)				35.8 (10.6)	
	**Ethnicity, n (%)**					
		Chinese		2 (11)		
		Malay		15 (83)		
		Other		1 (6)		
	**Religion, n (%)**					
		Islam		14 (78)		
		Christianity		1 (6)		
		None, atheist, or agnostic		3 (17)		
	**Relationship status, n (%)**					
		Single		6 (33)		
		Has a partner		12 (67)		
	**Housing status, n (%)**					
		Has a home		18 (100)		
		Homeless		0 (0)		
	**Completed high school, n (%)**					
		No		6 (33)		
		Yes		12 (67)		
	**PrEP^a^ knowledge and history, n (%)**					
		**Ever heard of PrEP**				
			No	2 (11)		
			Yes	16 (89)		
		**Ever used PrEP**				
			No	16 (89)		
			Yes	2 (11)		
		**Currently using PrEP (n=2)**				
			No	1 (50)		
			Yes	1 (50)		
		**Willing to use PrEP**				
			No	4 (22)		
			Yes	14 (78)		
	**Concerns about using PrEP, n (%)**					
		**Efficacy**				
			No	14 (78)		
			Yes	4 (22)		
		**Side effects**				
			No	7 (39)		
			Yes	11 (61)		
		**Safety**				
			No	13 (72)		
			Yes	5 (28)		
		**Cost**				
			No	6 (33)		
			Yes	12 (67)		
		**Convenience of acquiring the medication**				
			No	12 (67)		
			Yes	6 (33)		
		**Convenience of taking the medication**				
			No	13 (72)		
			Yes	5 (28)		
	**Recent behavior (past 6 mo), n (%)**					
		**Engaged in anal sex**				
			No	9 (50)		
			Yes	9 (50)		
		**Engaged in transactional sex**				
			No	14 (78)		
			Yes	4 (22)		
	**Smartphone and internet use, n (%)**					
		**Daily access to the internet**				
			No	1 (6)		
			Yes	17 (94)		
		**Smartphone as a primary device for internet use**				
			No	3 (17)		
			Yes	15 (83)		
**Stakeholders (n=11)**						
	Age (y), mean (SD)				43.0 (12.2)	
	**Ethnicity, n (%)**					
		Indian		3 (27)		
		Malay		8 (73)		
	**Occupation, n (%)**					
		Medical doctor		1 (9)		
		Clinic manager or administrator		5 (45)		
		Counselor		2 (18)		
		Social and outreach worker		3 (27)		
	**Type of health facility, n (%)**					
		Community-based organization		8 (73)		
		Clinic or hospital: government		2 (18)		
		Clinic or hospital: academic		1 (9)		
	**Facility offers HIV testing, n (%)**					
		No		1 (9)		
		Yes		10 (91)		
	**Involved in providing PrEP services, n (%)**					
		No		4 (36)		
		Yes		7 (64)		
	**Facility offers hormone therapy, n (%)**					
		No		10 (91)		
		Yes		1 (9)		

^a^PrEP: pre-exposure prophylaxis.

#### Transgender Women Participants

Among transgender women participants, the mean age was 35.8 (SD 10.6) years. The majority of study participants (15/18, 83%) identified as ethnic Malay. More than two-thirds of the participants (12/18, 67%) had completed high school, and all (18/18, 100%) reported having stable housing. Of the 18 participants, 6 (33%) reported being single, and 4 (22%) reported having engaged in sex work within the last 6 months. In terms of PrEP knowledge and history, most of the participants (16/18, 89%) were previously aware of PrEP for HIV prevention. Although only 11% (2/18) of the participants reported having ever used PrEP, most of them (14/18, 78%) indicated that they were willing to use PrEP. The most common concerns related to PrEP uptake included cost (12/18, 67%), side effects (11/18, 61%), efficacy (4/18, 22%), convenience of acquiring the medication (6/18, 33%), and convenience of taking the medication (5/18, 28%). Regarding smartphone and internet use, almost all participants (17/18, 94%) had daily access to the internet, with most of them (15/18, 83%) reporting their smartphone as the primary means of accessing the internet.

#### Stakeholders

Of the 11 stakeholders, 8 (73%) were Malay. Most of them were clinic managers and administrators (5/11, 45%), followed by social and outreach workers (3/11, 27%) and counselors (2/11, 18%). The stakeholders worked at community-based organizations (8/11, 73%), government clinics (2/11, 18%), and university clinics (1/11, 9%). Regarding facility services, 91% (10/11) offered HIV testing, and 64% (7/11) provided PrEP services. In addition, 9% (1/11) offered hormone therapy services, while the majority (10/11, 91%) did not.

### Qualitative Findings

In the codebook (Multimedia Appendix 2), four overarching themes emerged from the thematic analysis: (1) barriers to the uptake and use of PrEP, (2) feedback and request for additional smartphone app functional features, (3) feedback on the attributes of the app, and (4) suggestions for additional communication-related features.

#### Barriers to the Uptake and Use of PrEP

##### Stigma

Transgender women and stakeholder participants identified the stigma that targets transgender women, especially due to their visible transgender identity, as a significant barrier to seeking health care, including HIV prevention services. This stigma arises from sociopolitical structures, such as laws and policies that criminalize transgender identities coupled with unfair treatment that transgender women encounter within health care settings. Many participants emphasized that legal constraints and existing sociocultural values not only instill a deep sense of fear but also trigger shame, particularly when seeking sexual health services such as PrEP:

I don’t feel safe going to the hospital due to identity recognition, stigma, and discrimination, and people put down on me. When any TG [transgender] visits the hospital, people start to talk and look down on us. They always relate us to HIV, diseases, and things like this.

Transgender woman participant, FG3

[O]ne of the biggest challenges for trans women is the fact that they are considered unlawful in Malaysia. They live with that fear and shame. Even when they come to the clinic for PrEP, we see that they live with that fear.

Stakeholder, FG5

A participant recalled the experience of referring a PrEP-eligible peer to a government clinic for PrEP services:

One friend called me and asked if I knew any doctors or clinics providing HIV services. My friend went to the government clinic to test for HIV and get on PrEP. But the doctor did not prescribe PrEP...To them, prescribing PrEP is like encouraging us to have sex.

Transgender woman participant, FG2

A stakeholder participant who operates a primary care clinic that also offers HIV prevention and treatment services echoed these sentiments and explained how stigma in the form of dismissive attitudes and invasive questioning from clinic staff affects transgender women’s willingness to seek health care services:

Supposedly [transgender-]friendly clinics like mine, you know it [stigma and discrimination] still exists because, as you mentioned correctly, stigma does still exist, we are doing a lot to try and reduce it, but it still exists very strongly, actually...Sometimes staff can be a bit unfriendly or ask personal questions, which makes them [transgender women] uncomfortable.

Stakeholder, FG5

A transgender woman participant from Terengganu living in KL compared the experience of using HIV prevention and treatment services in their home state and the capital city. They noted that it is easier to seek services in KL due to the more open-minded and gender-affirming attitudes of health care providers, especially in private clinics:

I’m from Terengganu but live in KL. I definitely know how conservative the situation is in Terengganu. It’s very challenging for women like us. But for the KL [Kuala Lumpur] area, I can see that most of the private sector doctors are very open-minded. They are very understanding when it comes to trans women’s health care, including HIV and stuff. They have no problem and are open about that.

Transgender woman participant, FG3

##### PrEP Knowledge Gaps

Transgender women and stakeholder participants cited awareness of PrEP as a significant concern and a barrier to PrEP uptake and use among transgender women. Participants described how they and other transgender women in the community had limited knowledge about PrEP, its benefits, and where and how it can be accessed. Transgender women participants also stressed that much of the available information on PrEP was in English, creating a barrier for those who speak only Bahasa Malaysia:

We don’t have enough information on what PrEP is all about, its benefits over condoms, the cost, and where it is available. Is it only available at private or government hospitals, or can the pharmacy sell PrEP?

Transgender woman participant, FG4

We don’t have much information about PrEP, and again, a lot of information about PrEP is available in English. No one understands [English], yeah, they [transgender women] prefer a simple Malay language to make them understand.

Transgender woman participant, FG1

[The transgender] community is not aware of testing and PrEP medication, and some of them contact us [NGO] for support and education.

Stakeholder, FG6

Participants also shared recommendations for how to effectively build knowledge and interest in PrEP within the diverse communities of transgender women in Malaysia; for example, some transgender women underscored the importance of creating PrEP resources in local languages and providing community health workers (ie, case managers and social workers) with better training on HIV prevention and PrEP, including how to work effectively within transgender communities:

I see a lot of outreach workers and caseworkers who need to learn what PrEP is. They don’t know how to explain back [educate] to the community. We need to educate them more about PrEP and ways to communicate in ethnic dialects.

Transgender woman participant, FG1

##### Financial Barrier

Transgender women participants expressed concerns about the cost of PrEP medication and other PrEP-related expenses, including costs for physician consultations and laboratory tests, as a major barrier to both the uptake and continued use of PrEP. Many agreed with these sentiments and explained how a lack of financial resources affected transgender women’s ability to initiate or stay on PrEP:

The PrEP supply that I’m taking now costs RM 95 [∼ US $24] per bottle, and the doctor will give you a three-month prescription. Some of our sisters think it is too costly. They [transgender women] will be on PrEP for 3 to 6 months, then suddenly stop due to financial issues.

Transgender woman participant, FG2

Most stakeholders echoed these sentiments and elaborated on how the lack of subsidized or free PrEP for transgender women in government-run primary care clinics prevented transgender women from starting and staying on PrEP:

One of the barriers is the cost of PrEP. We can’t offer trans women free PrEP as we don’t have it in our [government’s] primary care formula. PrEP is only free for serodiscordant married couples, then the [HIV-negative] lady gets it for free, or the husband.

Stakeholder, FG5

##### Alternative HIV Prevention Methods

Transgender women participants were found to be skeptical of PrEP in general, which emerged in 3 unique ways. First, several transgender women participants noted that there were alternative, less expensive preventive methods, such as condoms, that conferred protection against HIV as well as STIs:

It [PrEP] only protects us from HIV, not other STIs. To avoid wasting [money] on the cost, we prefer to use condoms, which have the same function as PrEP. Once you use condoms, you are safe.

Transgender woman participant, FG2

PrEP is so expensive, and this is why I use condoms. Better to use condoms because they are cheap.

Transgender woman participant, FG1

Second, several transgender women had concerns over the efficacy of PrEP in reducing HIV transmission:

I am not 100 percent convinced that it [PrEP] works effectively to prevent HIV infection.

Transgender woman participant, FG4

Several stakeholders also confirmed that transgender women participants preferred to use condoms over PrEP because they deemed condoms safe and effective:

[T]rans women still believe the effectiveness of using condoms is better than using PrEP. Making them use PrEP is still a long way to go because, like I say, what they believe will be safe for them would be using condoms instead of PrEP.

Stakeholder, FG6

Third and last, a few transgender women participants cited the burden of maintaining regular adherence to daily PrEP intake as a potential barrier, and some mentioned that transgender women chose condoms over PrEP to avoid the daily pill burden:

[I]t’s a bit challenging because when sister [transgender women] meets me, they tell me that they do not follow the doctor’s advice on PrEP timing. They just eat [swallow] it, and then it is already wrong in terms of taking the daily PrEP correctly.

Transgender woman participant, FG3

In terms of PrEP, one thing is the lack of monitoring of the PrEP intake [adherence]. Sometimes, young trans women that take the PrEP don’t have the consistency [to adhere to PrEP]. They would also forget themselves, okay, so that is one of the issues, and they choose condoms.

Transgender woman participant, FG2

##### PrEP Accessibility

Transgender women and stakeholder participants stated that most PrEP clinics were available in densely populated urban centers, such as KL. For transgender women in suburban or rural areas, accessing PrEP often involved long travel times to reach the PrEP clinics:

One of the major barriers to using PrEP I see is availability, especially in rural areas, so many trans folks are excluded because the location and service providers are out of their reach.

Transgender woman participant, FG3

PrEP is only available in a certain location, like in KL [Kuala Lumpur], but how about if you’re in Johor Bahru or if you’re in Kota Bahru? Where do we go if you want [PrEP]?

Stakeholder, FG6

A stakeholder described how it was difficult for transgender women, compared to transgender men or cisgender MSM, to travel and access PrEP across cities due to the existing social and structural barriers:

I’m in PJ [Petaling Jaya], so my clients, especially trans men and MSM, seem very comfortable traveling from KL [Kuala Lumpur] and taking the services [PrEP]. But you know, it’s a different social structure for trans women to come out and travel to my clinic, so distance is an issue.

Stakeholder from FG5

##### Perceptions About the Adverse Effects of PrEP

Several transgender women described having concerns about the potential side effects of PrEP, including that the use of daily oral PrEP might have adverse effects on their overall health and well-being:

When we consume PrEP, it will affect the liver and kidneys. Is that right?

Transgender woman participant, FG4

A stakeholder working in a community-based organization also expressed that their transgender women clients are concerned about potential interactions or the combined effects of being on PrEP and hormone therapy:

Something that trans gender will want to know is if you take an HRT shot and be active in PrEP, what are the side effects? I mean, what is the side effect of taking both?

Stakeholder, FG6

#### Theater Testing of the HealthMindr App for Adaptation Among Transgender Women in Malaysia

Theater testing of the HealthMindr app during the FG sessions yielded essential feedback on the format, content, and features for potential adaptations to the needs of transgender women in Malaysia. Important suggestions included participants’ recommendations on the app’s attributes, features, and the addition of more functional and communication features.

#### Privacy and Confidentiality

Several participants suggested giving extra attention to protecting the privacy and confidentiality of app users’ data:

The database should be very confidential like if new transgender folks’ kind of like registered with the app, their details should be like confidential...so this is very important, especially for sex workers, because any of this should not be out there and like be known to government and others that might harm you.

Transgender woman participant, FG1

Clinical stakeholders also reiterated the need for app developers to create a comprehensive data protection plan and implement security measures to build trust and comfort among transgender women users of the app:

We also need to instill confidence in the security of the mobile app by indicating their [app users] information is very safe, and they [app developers] are not going to share it with anybody. I think this [information] needs to be highlighted in the profile place, really.

Stakeholder, FG5

#### Modifications to the Look and Feel of the App

Many participants suggested changing the color of the app. They preferred some color schemes from the transgender flag. Most transgender women participants suggested using pink or purple or a combination of the 2 to increase the app’s appeal and improve user retention as mentioned by the following transgender woman participants from FG2:

Participant 1: Purple, pink, or something pastel.

Participant 2: Pastel is not so interesting. Purple is fine.

Participant 1: Purple, yes, that is why I said just now, like rainbow colors, so when people see it, they will know, oh, this app is for us and use it regularly.

Participants also suggested developing a simple and easy-to-use interface for all age groups:

The user interface has to be user-friendly, like everyone like, no matter what your age right, like everyone can use it, that should be like the perfect app for like all the transgender folks, yep.

Transgender woman participant, FG1

#### Ensuring Multilingual Functionality

Several participants (both transgender women and stakeholders) suggested making the app multilingual to reach diverse transgender women in Malaysia. Their suggestion was to offer users the choice of language, enhance accessibility, and fulfill the diverse linguistic preferences within the transgender women community in Malaysia:

We live in a multiracial country. Why doesn’t the app get translated into Bahasa Malaysia? And we can use our preferred language for the app.

Transgender woman participant, FG2

Participants suggested that the app’s theme and avatar on the profile page be customizable to enhance user ownership:

I prefer, like, I came across with some app where they let the users customize their avatar, like the skin color, different hair color, that kind of thing, so I feel like properly represented. Let this app user feel like they are appropriately represented.

Transgender woman participant, FG3

#### Adding New Features and Functions

Participants provided feedback on the HealthMindr app’s features. New functions that could cater to the needs of transgender women and break the existing barriers to using HIV prevention and treatment services were also requested. The participants proposed expanding appointment booking features beyond HIV prevention services to include other primary health care services to motivate transgender women to care for their health:

[M]aybe we can add appointments on general health testing like checking blood sugar, cholesterol, and blood pressure as trans women prefers. It can attract them as these diseases are associated with hormone taking. I would love it if you could include these general health tests in the app. If we only include HIV services, the client will feel scared, especially the trans women community.

Transgender woman participant, FG2

Participants also suggested adding a telehealth-style e-consultation feature so that transgender women could consult healthcare providers and experts from the transgender community before starting PrEP, hormone therapy, and other medications through the app. Participants also proposed that both chat and video options could be included for the e-consultation so that the users could choose their preferred mode:

I prefer to have an e-consultation feature in the app with chat, audio, and video options, and it could be better if it’s someone from our community specifically for hormones. Of course, the doctors know something about medicine and stuff, but they don’t really know how to go through hormones with the correct routines and stuff to transition. So, I think someone, community members, who are an expert is much more valuable in the matter of hormones.

Transgender woman participant, FG3

Two stakeholders also discussed the e-consultation features, expressing strong interest in providing e-consultation services via the app so that transgender women can avoid having to travel to the health care facility:

I am always ready to provide virtual care through the app. This will cut down the need for trans women to come to any facility to get health care services.

Stakeholder, FG5

Participants also suggested including an e-pharmacy feature that allows them to fill prescriptions (eg, PrEP, antiretroviral therapy, hormones) and order medical products (eg, HIV self-test kits). In addition, participants discussed offering different delivery options so that users could choose the most convenient one:

Actually, you can include an online pharmacy feature, where you can order without entering so much of your personal details, just the name and the online pharmacy will post or deliver the medication to the client. Client can choose to take it from the runner, put it in the postbox, or self-pickup it up from the Pharmacy.

Transgender woman participant, FG4

Participants appreciated the *Medicine Manager* feature of the HealthMindr app. They felt that the feature would help remind users when to take their PrEP and their hormones, as well as when to refill their prescriptions:

It is a very interesting app, seriously. You can add the name of your medicines, how many times you have to take them, and stocks, and it reminds you daily and sends a refilling reminder. I love this innovation. It will be very easy to remember PrEP medication and hormone shots.

Transgender woman participant, FG3

One of the most appreciated features of the app was the mood tracker. Several participants found this feature the most helpful because they believed that transgender women often required mental health and psychological support:

The best feature of the app is the mood tracker. I think because every day, we have a lot of issues so then we can track. I think this is the best part. At least you can see how the improvement is because normally, the mood will reflect our mental health, right?

Transgender woman participant, FG4

Participants also provided a few recommendations to improve the mood tracker feature. A few suggested adding notifications to prompt users to log their mood:

App users actually have to come to this, click on it, and add their mood there right. Can they then open the app so that the question is automatically asked? I am not so IT savvy, so I do not know whether this can be done, but once an app is open, it should ask them how you are feeling today.

Stakeholder, FG5

Furthermore, participants suggested including a list of mental health practitioners who could offer help to transgender women who needed it:

[T]here is information on mental health through a mood tracker, but what is next? The important thing is that the app should be able to suggest any affiliated mental health practitioner in case one needs them.

Transgender woman participant, FG4

Participants responded favorably to the resource features on HIV and PrEP; they felt that they did not have adequate information on PrEP and hormones and that the feature would provide helpful information. They also suggested adding a frequently asked questions section:

Add a frequently asked questions section, where answers are already given in the app, like general information regarding hormones, their side effects, long-term effects, and things like that, which will be very good.

Stakeholder, FG5

Moreover, participants recommended adding simple graphics, images, and audiovisual aids as a resource so that all age groups could benefit from them:

A section that you click like a video, so some people prefer to look into a video. Maybe we can create a video and upload it explaining PrEP and HIV in the Malay version.

Transgender woman participant, FG1

For the older generation, we need to have a video right and at the same time images and graphics in a simple language.

Transgender woman participant from FG1

Both transgender women and stakeholder participants discussed the importance of a feature suggesting nearby transgender-friendly clinics that provide HIV prevention and hormone therapy care services:

If it’s not in this app, my suggestion is to add the locations and contact details of the clinics in the app that are definitely queer-friendly, very respectful, and knowledgeable about treating trans folks.

Transgender woman participant, FG1

[A]nd then, where to get the hormone, where can [one] get transgender-friendly services, I think that’s probably something you can add. I’m sure there’s a list of [transgender-]friendly clinics, so maybe you can add that.

Stakeholder, FG6

Participants mentioned the importance of including the location and details of the transgender-friendly clinics and asked to include the feature in the app by giving an example:

For example, on the app, if we choose our location in Kuala Lumpur, then it should list the location to get hormones/pharmacy that provides PrEP, HIV self-test kits, and a home visit from a private doctor or NGO.

Transgender woman participant, FG2

A clinical stakeholder suggested including details of ongoing promotions of clinical services within the locator feature:

Now, just thinking, if you have a kind of a list of friendly clinics, and maybe you can also include the details of the promotion that the clinic is giving, like you know if you go in for like testing at a specific time, a certain percentage of the discount.

Stakeholder, FG5

#### Suggestion for an Interaction-Enabled Social Communication Feature

Participants expressed interest in having several options to communicate with other transgender women to share their experiences and get their peers’ support, mentorship, and coaching. They also recommended including a feature where they could communicate with legal advisors while remaining anonymous:

I would suggest we do have a corner to get peer support from sisters so that you know some trans women prefer to remain anonymous and are willing to share their stories and seek advice.

Transgender woman participant, FG2

You know, it is really important for us to have legal support. We face a lot of legal challenges, and sometimes things can get bad when we go out. We need to know how to protect ourselves, especially if our sisters get arrested... It would be great to have a feature where we can communicate with legal advisors.

Transgender woman participant, FG3

We can also talk about how to get a job, right? And inside the app, maybe we can put in how to start a business and then how the trans business can work together and connect. And we need all the inspiration from inside and outside. I believe we have a lot of inspirational and successful young trans women.

Transgender woman participant, FG2

## Discussion

### Principal Findings

This study examined barriers to PrEP uptake among transgender women in Malaysia, as well as their preferences for mobile app features that could facilitate access to HIV prevention and gender-affirming care services. The identified PrEP barriers included high stigma and discrimination, limited PrEP knowledge, high costs, accessibility concerns, alternative prevention methods, and perceived adverse effects. During theater testing of the app, participants highlighted the importance of certain attributes and functions, such as a user-friendly interface, app language of choice, customization options, user feedback mechanisms, appointment booking, e-consultation, e-pharmacy, medicine tracking, mood tracking, resource provision, and service site locator functions, as well as communication features, such as peer support, live chat, and discussion forums.

Experience of stigma from health care providers was commonly cited as a barrier to accessing HIV prevention services, including PrEP [9,16,25]. Consistent with the findings of other studies, many transgender women participants in our study reported hesitancy in accessing public health care facilities for HIV testing and PrEP due to transgender visibility–and identity–related stigma, compounded by legal prejudices and misconceptions among health care providers who believed that PrEP encourages sex and frequently associated transgender women with HIV [41,42]. Participants’ preferences for mobile app features, such as discreet delivery of HIV self-test kits, PrEP medication, and hormone doses to a preferred location, could potentially create a more convenient and confidential experience. Integrating features such as web-based appointments and e-consultation with transgender-friendly physicians could contribute to a less stigmatizing environment where transgender women can feel more at ease and would not feel judged. Similarly, collaborating with organizations serving transgender women to provide value clarification and awareness training [43] for health care providers delivering HIV prevention services is crucial and sustainable, given that transgender women experience stigma in government health care settings, which is identified as a barrier to PrEP use. Training programs should emphasize cultural competence and sensitivity, educating providers about the unique health needs of transgender women and the critical role of PrEP in HIV prevention. Such initiatives are vital to foster a supportive and inclusive health care environment, ultimately reducing barriers to accessing essential health services.

The lack of PrEP awareness and knowledge among transgender women, providers, and HIV outreach workers has been cited as one of the most common barriers to PrEP uptake [44,45]. Transgender women participants wanted comprehensive information on PrEP, its safety, efficacy, and side effects, especially the potential adverse effects of using hormones as part of their gender-affirming care services [46-48]. Several studies have demonstrated that multilingual mobile apps can effectively enhance HIV- and PrEP-related knowledge, behavior, and access to services, with strong acceptability among transgender women [34,49,50], underscoring the relevance and importance of continuing to explore such interventions. Moreover, improved education and training for health care providers and outreach workers could enable them to disseminate accurate and up-to-date information about PrEP and gender-affirming hormones to transgender women, helping them to make informed choices [51].

The cost of PrEP was a major concern among transgender women participants, highlighting the economic marginalization faced by this population. This finding aligns with previous research on PrEP barriers in transgender women [52,53]. While PrEP-related cost remains a concern for transgender women, the Malaysian government’s recent initiative to provide PrEP for free in a few government clinics is a positive development. However, backlash from Malaysian religious groups regarding providing PrEP for sexual and gender minority populations as well as long hospital waiting periods remain issues [22,26]. To enhance the accessibility and acceptability of the free PrEP program for transgender women in Malaysia, it is crucial to expand the services nationwide. In addition, integrating mHealth interventions such as web-based PrEP consultations and delivery of HIV self-testing kits and PrEP medication in the preferred location minimizes the need for in-person visits, reduces travel and other out-of-pocket expenses, and could potentially enhance convenience and overall PrEP accessibility [54].

A notable preference for condoms over PrEP among transgender women was linked to doubts about PrEP efficacy, cost concerns, adherence issues, and accessibility challenges [52,53]. The preference for condoms may be rooted in historical familiarity, cost-effectiveness, accessibility, and dual protection against various STIs, including HIV. To increase PrEP knowledge and acceptability among transgender women, efforts should focus on providing PrEP information, education, and communication specific to transgender women through different media; for instance, participants in this study preferred PrEP information in various ethnic languages and audiovisual forms, which has effectively increased transgender women’s knowledge in other settings [34,49,50]. Similarly, PrEP should be part of a comprehensive plan that includes condoms and other HIV and STI prevention methods [46,55]. Long-acting injectable PrEP could be explored as a viable option for those who have challenges remaining adherent to daily oral PrEP [56].

Our findings support the notion that transgender women value and trust the *sisters circle*, a social network and community of transgender women. Transgender women cited several times that they would feel comfortable sharing and receiving important sources of information on PrEP and gender-affirming care services, among others, that provide resources for their health and well-being from their peer circle. The mobile app, with various communication features, such as discussion forums and live chats, could potentially create a safe space for the transgender women community to learn and share experiences with their peers about HIV, PrEP, and gender-affirming care services. Internet-based spaces and peer support could potentially neutralize internalized stigma and improve HIV-related outcomes in transgender women [57-59].

The participants expressed significant concerns regarding the privacy and confidentiality of their data when using the app. Specifically, similar to Malaysian MSM participants, they were concerned about potential data leaks to governments or third parties [60,61]. As a proactive solution, the participants recommended the incorporation of a robust data protection plan for app users. The findings suggest that while developing the app, it is important to have high-end privacy and confidentiality features to make it a trustworthy platform for transgender women users.

### Limitations

This study has limitations. First, participants were recruited via convenience sampling through social media platforms and partner NGOs. Thus, they are only representative of some of the transgender women in Malaysia, and the views of transgender women without access to smartphones were not taken into account. Second, participants did not have the opportunity to download and use the HealthMindr app; therefore, they could not provide detailed feedback on the mobile app’s specific features and functionality. Finally, it is important to note that, although participants may express their willingness to use a particular feature, actual adoption may differ. Therefore, it is necessary to evaluate the real-world use.

### Conclusions

The findings from the FGs revealed key barriers to PrEP uptake and use among transgender women in Malaysia. The findings also provide detailed recommendations for successfully adapting the HealthMindr app to the context of Malaysian transgender women, with a potential solution for delivering tailored HIV prevention and gender-affirming care services. Mobile apps with the suggested features have the potential to overcome some of the barriers to PrEP and promote HIV prevention efforts among transgender women in Malaysia. It is crucial to incorporate these insights into developing the alpha version of the app and to test it with transgender women to tailor it to their specific needs. Moreover, further research is needed to determine the long-term effectiveness of this intervention and explore other potential barriers to PrEP access and adherence among transgender women in Malaysia.

## Appendix

Multimedia Appendix 1 Focus group guide.

Multimedia Appendix 2 Codebook from thematic analysis.

## Abbreviations

FG: focus group
HRT: hormone replacement therapy
KL: Kuala Lumpur
mHealth: mobile health
MSM: men who have sex with men
NGO: nongovernmental organization
PrEP: pre-exposure prophylaxis
STI: sexually transmitted infection

## Data Availability

The data sets generated and analyzed during this study are not publicly available due to the sensitive nature of the information in the transcripts and the potential for severe ethical, legal, and social consequences if confidentiality is breached, but redacted excerpts are available from the corresponding author on reasonable request.
