# Supplementary material for: Adaptation of a Theory-Based Mobile App to Improve Access to HIV Prevention Services for Transgender Women in Malaysia: Focus Group Study
Source: JMIR Form Res. 2024 Aug 13;8:e56250. doi: 10.2196/56250 (PMC11350319; doi:10.2196/56250)
Supplement: Multimedia Appendix 1 [file formative_v8i1e56250_app1.docx]

**Appendix 1: Focus Group Guide**

**Topic 1: PrEP Barriers/Challenges**

1. What makes getting on (and staying on) PrEP difficult or challenging for trans women in Malaysia?

**Topic 2: “There’s an App for That!” – How can an app help you with HIV prevention that includes PrEP?**

1. If you could design your own app to help you get regularly tested for HIV and help you get PrEP, what would that app do? (Ask participants to be specific to HIV testing and PrEP)

**Topic 3: In addition to HIV testing & PrEP, what else should this app do? Other features?**

1. Gender-affirming medical care support/services
2. Gender-affirming counselling / mental health support
3. Employment / Economic opportunities

**Topic 4: Tour of the Healthmindr app & Feedback**

**App’s functions and features**:

1. What did you most like about the Healthmindr app’s functions?
2. What did you least like about the Healthmindr app’s functions?
3. What did you think of the following features:
   1. Avatar/profile photo feature?
   2. Points system (earn and redeem points)?
   3. Messaging system (chat with the app team or your clinic)
   4. Promotion of PrEP supply
   5. Others….
4. Is there any function in Healthmindr that you would suggest we remove/delete in the new app for trans women?
5. Is there any function you would suggest we add/include in the new app for trans women (that is not in Healthmindr)?

**Look and feel of the app:**

1. What did you most like about the look and feel of Healthmindr?
2. What did you least like about the look and feel of Healthmindr?
3. If you could change anything about the color scheme/colour pallet, what would you change?
