# Supplementary material for: Adaptation of a Theory-Based Mobile App to Improve Access to HIV Prevention Services for Transgender Women in Malaysia: Focus Group Study
Source: JMIR Form Res. 2024 Aug 13;8:e56250. doi: 10.2196/56250 (PMC11350319; doi:10.2196/56250)
Supplement: Multimedia Appendix 2 [file formative_v8i1e56250_app2.docx]

**Appendix 2: Codebook from thematic analysis**

| **Root code** | **Child code** | **Description** | **Trans women** | **Stakeholders** |
| --- | --- | --- | --- | --- |
| Barriers to proper PrEP use | Stigma | Stigma due to being visibly trans and stigma associated with PrEP in the health care setting; some medical providers believe that PrEP encourages people to have sex | ✓ | ✓ |
|  | Limited knowledge of PrEP | Inadequate promotion of PrEP information, limited knowledge about PrEP, its benefits and side effects | ✓ | ✓ |
|  | Expensive | Not in the primary care formula, only free for serodiscordant married couple | ✓ | ✓ |
|  | Accessibility | An individual's access to PrEP due to geographic location: traveling long distances to clinics, and limited availability of PrEP in rural areas | ✓ | ✓ |
|  | Alternatives prevention methods | Availability of cheaper options like condoms that offer protection against other STIs | ✓ | ✓ |
|  | Perceived adverse effects | Concerned about the side effects of PrEP, especially those taking hormones. | ✓ | ✓ |
| **App features preference** | | | | |
| Feedback and request for functional features | Appointment booking | Be able to make clinic appointments. | ✓ | ✓ |
|  | e-Consultation | Connect with health care providers and get medical advice within the app. | ✓ | ✓ |
|  | Online pharmacy | Online order of medication (HIV self-testing kit, ART, PrEP, hormones, and other medicines) for home delivery, self-pickup, or drop in the postbox. | ✓ | ✓ |
|  | Medication tracker | Reminder and tracking of medicines (ART, PrEP, hormonal, or any other medications that the user is taking | ✓ |  |
|  | Mood tracker | Mood assessment and support services for those who need psychological support. | ✓ | ✓ |
|  | FAQ/ resources sections | Information and resources on HIV self-test kits, HIV testing, PrEP, hormone use and possible side effects, gender-affirming care services, and mental health, trans friendly legal services info | ✓ | ✓ |
|  | Service site locations | A feature that suggests nearby clinics, especially trans women friendly ones, with contact details and a map. | ✓ | ✓ |
| Feedback on the attributes of the app | Private and secure | Data security from unauthorized access; methods in which the app developers can ensure no data leakage | ✓ | ✓ |
|  | Visually appealing | Images and videos to explain information presented over written text, rainbow colors | ✓ | ✓ |
|  | User friendly | Simple and easy-to-use interface for all age groups | ✓ | ✓ |
|  | Language of the app | Options to choose a language specifically between Malay and English | ✓ | ✓ |
|  | Customizable theme, color, and avatar | Users to choose different background themes and colors and could be able to customize the avatars. | ✓ | ✓ |
| Suggestions for additional communication features | Peer support group/discussion forum | A feature that allows communication with the trans women community for problems solutions, coaching/mentoring and encouragement, and legal advice | ✓ | ✓ |
|  | Live chat | Communication with multidisciplinary healthcare providers and peers for medical advice within the app | ✓ |  |
